# Using the Major Components (Cellulose, Hemicellulose, and Lignin) of *Phyllostachys praecox* Bamboo Shoot as Dietary Fiber

**DOI:** 10.3389/fbioe.2021.669136

**Published:** 2021-03-31

**Authors:** Jinlai Yang, Liangru Wu, Huimin Yang, Yanhong Pan

**Affiliations:** ^1^China National Bamboo Research Center, Hangzhou, China; ^2^Key Laboratory of High Efficient Processing of Bamboo of Zhejiang Province, Hangzhou, China; ^3^Key Laboratory of Resources and Utilization of Bamboo of State Forestry Administration, Hangzhou, China

**Keywords:** bamboo shoot powder, preparation, dietary fiber, mice, bio-application

## Abstract

Bamboo shoots are a renewable and abundant biomass containing cellulose, hemicellulose, and lignin. Although many studies have explored the applications of each of these components in the preparation of biochemicals and biopolymers, few studies have evaluated the utility of these components as a dietary fiber supplement. In this study, a powder consisting of the main components of bamboo shoots (cellulose, hemicellulose, and lignin) was prepared from fresh *Phyllostachys praecox* shoots and characterized by scanning electron microscopy, infrared spectroscopy, and X-ray diffraction. To evaluate the potential utility of these components as a dietary fiber supplement, we conducted an experiment in which this powder was supplemented in the diet of mice for 7 weeks. The experiment included three diet groups (*n* = 10/group): a low-fat control diet (LFC), high-fat diet (HFD), and high-fat diet with bamboo shoot powder (HFBSP). Compared with HFD mice, the body weights of LFC and HFBSP mice were lower, indicating that the addition of bamboo shoot powder could reduce the weight gain associated with the HFD. Bamboo shoot powder supplementation could also reduce the levels of triglycerides (TG), blood glucose (GLU), total cholesterol (CHOL), high-density lipoprotein (HDL-C), and low-density lipoprotein (LDL-C) in HFD mice. The fat histology images indicated that obesity was alleviated in HFBSP mice, and the liver histology images indicated that the addition of bamboo shoot powder to the HFD could reduce the risk of fatty liver disease. The addition of bamboo shoot powder to the HFD might also improve the gut microbiota of mice. Thus, the major components of bamboo shoot powder (cellulose, hemicellulose, and lignin) could be used as beneficial natural additives in the food industry.

## Introduction

Vegetables are important for a balanced and healthy diet (Bvenura and Sivakumar, 2017). However, foods with high calories and fat are also frequently enjoyed in high-income countries (Hunter et al., 2019) because the consumption of healthy vegetables alone is insufficient to meet caloric needs. Bamboo is a useful perennial woody grass in temperate and tropical regions of the world (Huang et al., 2019b; Lin et al., 2019; Zheng Y. et al., 2021); shoots and wood are the two main products of bamboo. The shoots of bamboo are edible and rich in dietary fiber, protein, amino acids, polysaccharides, polyphenol, and minerals (Singhal et al., 2013; Huang et al., 2018; Dong et al., 2020).

An increasing number of studies have examined bamboo shoots. Cellulose nanocrystals and cellulose nanofibrils can be prepared from bamboo shoots by acid hydrolysis, which can be used in the fields of composite materials, drug delivery, and emulsifiers (Wijaya et al., 2019; Huang et al., 2020; Wang et al., 2020). Water-insoluble bamboo shoot dietary fiber was used as a plant food particle stabilizer to produce stable oil-in-water (O/W) Pickering emulsions (He et al., 2020). A distinctive antifungal protein (20 kDa) was isolated from fresh bamboo shoots (Wang and Ng, 2003). The bioactive constituents of bamboo shoot (*Bambusa balcooa*) extract could cause an imbalance in the oxidative status of thyrocytes, which impairs the action of hormone-synthesizing elements at the cellular and molecular level (Sarkar et al., 2019; Zheng L. et al., 2021). Polysaccharides extracted from bamboo shoots (*Chimonobambusa quadrangularis*) by ultrasonic-assisted extraction (Chen et al., 2019) or accelerated solvent extraction (Chen et al., 2018) show high antioxidant activity. In addition, the compositions in bamboo can be used to prepare the fluorescent composition to image cells and organs in different fields (Huang et al., 2019a; Su et al., 2019; Pei et al., 2020; Chen et al., 2021). A novel fluorescent composition was successfully isolated from winter Moso bamboo shoots and used to image human hepatocellular carcinoma cells (HepG2) (Yang et al., 2019).

A bamboo shoot diet was shown to decrease serum total cholesterol, low-density lipoprotein, and the atherogenic index (Park and Jhon, 2009). Four-week administration of bamboo shoot shell fibers alleviated diabetic syndrome in mice (Zheng et al., 2019). Mice that consumed a diet supplement with bamboo shoot shell (*Leleba oldhami* Nakal) had lower body weight gain (2.84%), total cholesterol (31.53%), triglyceride levels (21.35%), and low-density lipoprotein cholesterol (31.53%) and higher high-density lipoprotein cholesterol (37.6%) (Luo et al., 2017). Mice that consumed a diet supplemented with two bamboo shoot fibers (BSFs) from *D. hamiltonii* and *D. latiflorus* had lower body weight gain, lower levels of fasting glucose and insulin, and lower values of glucose area after 13 weeks, suggesting that BSFs can control insulin resistance and reduce the risk of type 2 diabetes (Li et al., 2018).

Although bamboo shoots are delicious and healthy vegetables, especially when consumed fresh, the freshness of bamboo shoots is difficult to maintain because of lignification (Luo et al., 2012). In addition, the bioavailability of nutrients and other extracts from different biomass depends on a variety of factors, such as compositions, structures and functional groups (Nair and Augustine, 2018; Yu et al., 2020; Tao et al., 2021). In this paper, we prepare a bamboo shoot powder from fresh bamboo shoots (*Phyllostachys violascens*), which contains different compositions of cellulose, hemicellulose, and lignin. The health effects of the powder were assessed by conducting an experiment in which the powder was supplemented in the diet of mice for 7 weeks. Aside from providing a new edible product of bamboo shoots, the results of our study highlight the potential benefits that the functional components of bamboo shoots could provide as a dietary supplement.

## Materials and Methods

### Materials and Instruments

Fresh bamboo shoots (*Phyllostachys violascens*) were obtained from Jianou, Fujian, China. All animal experiments were conducted with the approval of the Institutional Animal Care and Use Committee (IACUC) of Nanjing Medical University. Female mice were purchased from the Model Animal Research Center of Nanjing University. The lab mice maintenance diet was purchased from Jiangsu Synergetic Pharmaceutical Bioengineering Co., Ltd. (Nanjing, China). Reagents and solvents were purchased from commercial suppliers and used without further purification. The bamboo shoot powder was examined by a SU8010 scanning electron microscope. Infrared (IR) spectra were recorded on a Nicolet 380 FT-IR infrared spectrometer. XRD data were obtained using a Rigaku Ultima IV X-ray diffraction instrument. High-speed double-channel pulping was carried out by a GFM-DFP-200 engine.

### Preparation of Bamboo Shoot Powder

Bamboo shoot powder was prepared from fresh Lei bamboo shoots (*Phyllostachys praecox* shoots). The fresh shoots were cut into pieces after removing the peel and washing with water. The shoot pieces (200.0 kg) were crushed to smaller watery particles (<3 mm) through a high-speed double-channel pulping engine (1.0 mm, 0.6 mm), which generated a beating suspension. Finally, a bamboo shoot powder (17.2 kg, <80 mesh) was obtained after the beating suspension was dried by a spray-drying method. The powder had a unique aroma and nutrients; the yield was 8.6%, and the water content was 3.2%.

### Mice and Diets

After acclimatization (2 weeks), the mice were fed three types of diets for 7 weeks: a low-fat control diet (LFC), high-fat diet (HFD), and high-fat diet with bamboo shoot powder (HFBSP); the ingredient composition of the experimental diets is shown in Table 1. The mice were maintained on a 12 h light/12 h dark cycle at a temperature range of 20–24°C in a humidity-controlled environment.

**TABLE 1:** Ingredient composition of the experimental diets.

Ingredients	LFC	HFD	HFBSP
Maintenance diet (g)	100.0	57.6	57.6
Lard oil (g)	–	15.0	15.0
Sucrose (g)	–	20.0	20.0
Casein (g)	–	5.0	5.0
Cholesterol (g)	–	1.2	1.2
Sodium cholate (g)	–	0.2	0.2
Calcium bicarbonate (g)	–	0.6	0.6
Stone powder (g)	–	0.4	0.4
Bamboo shoot powder (g)	–	–	1.0

*LFC, low-fat control diet; HFD, high-fat diet; HFBSP, high-fat diet with bamboo shoot powder.*

### Serum Lipid Analysis

Samples of blood were obtained from live female mice, and the serum was isolated by clotting and centrifugation. The serum levels of triglycerides (TG), blood glucose (GLU), total cholesterol (CHOL), high-density lipoprotein (HDL-C), and low-density lipoprotein (LDL-C) were determined using a Hitachi 7100 blood automatic biochemical analyzer (Li et al., 2002). Serum levels of CHOL and TG were tested by enzymatic assays. HDL-C levels were measured after precipitation of apolipoprotein B-containing lipoproteins by dextran sulfate/magnesium chloride. LDL-C levels were determined from the Friedewald formula.

### Histology and Staining

After dehydration and washing, fat and liver were paraffin-embedded. Each tissue specimen was made into paraffin sections (5 Mm). The sections were stained with hematoxylin-eosin (HE), and the staining images were analyzed on an Olympus microscope.

### Gut Microbiota Profiling

Fecal samples were collected from live female mice and stored at –80°C; DNA was then extracted by the Omega Biotek E.Z.N.A. Stool DNA kit. PCR was used to amplify variable regions (3 and 4, V3-V4) of the 16S rRNA gene with the modified primers 338F (ACTCCTACGGGAGGCAGCAG) and 806R (GGACTACHVGGGTWTCTAAT). The gut microbiota data were collected using previously described methods (Schloss et al., 2009; Segata et al., 2011; Fan et al., 2020).

## Results and Discussion

### Bamboo Shoot Powder

**FIGURE 1:**
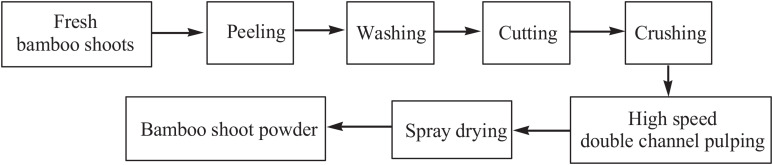
Preparation process of shoot powder from fresh Lei bamboo shoots.

The bamboo shoot powder was obtained from fresh Lei bamboo shoots (Figure 1). The powder had a unique aroma, dietary fiber, and abundant nutrients because the fresh shoots were rapidly processed. The main ingredients of the shoot powder were cellulose (12.47%), hemicellulose (12.94%), lignin (20.24%), and protein (21.18%); the total dietary fiber content was 45.65%.

**FIGURE 2:**
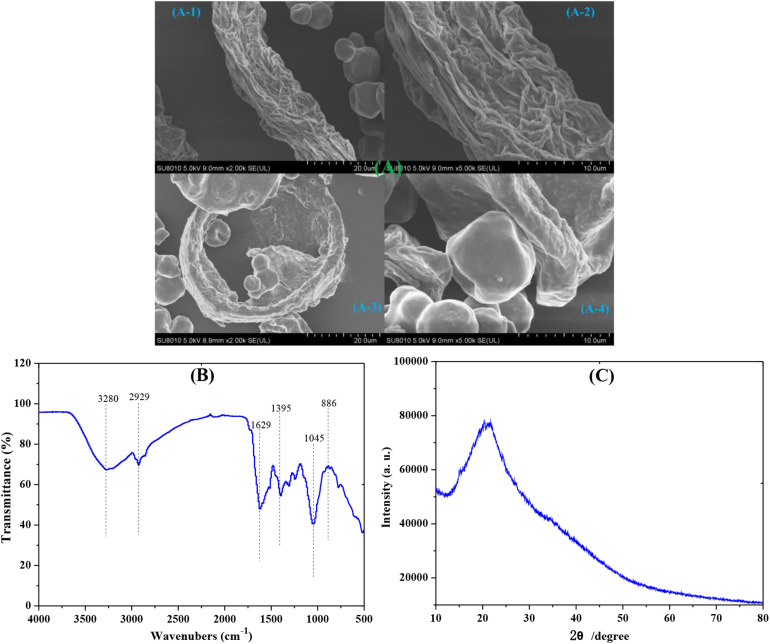
SEM **(A)**, IR **(B)**, and XRD **(C)** of bamboo shoot powder.

**FIGURE 3:**
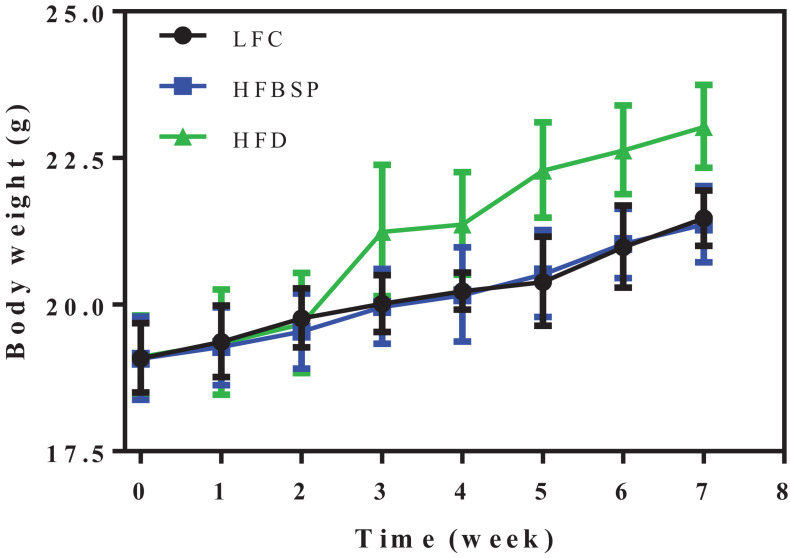
The effect of bamboo shoot powder on the body weight of mice fed a HFD for 7 weeks. Data are means ± SEM, *n* = 10/group.

**FIGURE 4:**
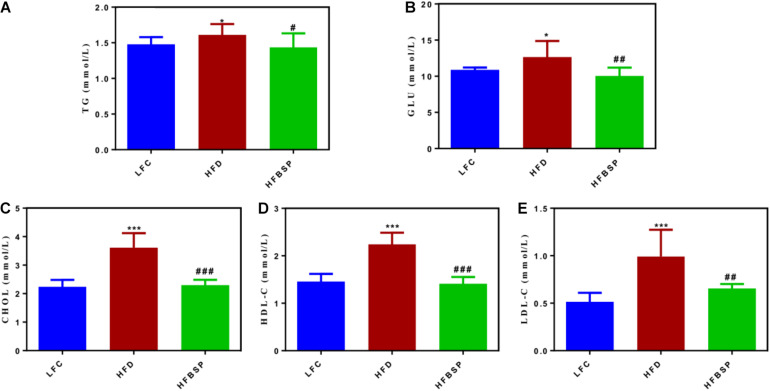
The effect of bamboo shoot powder on the biochemical parameters of TG **(A)**, GLU **(B)**, CHOL **(C)**, HDL-C **(D)**, and LDL-C **(E)** of mice fed a HFD for 7 weeks. Data are means ± SEM, *n* = 10/group. *p < 0.05, ***p < 0.001, vs. LFC group. ^#^*p* < 0.05, ^##^*p* < 0.01, ^###^*p* < 0.001, vs. HFD group.

The bamboo shoot powder was also characterized by SEM, IR, and XRD (Figure 2). The bamboo shoot powder was mixed with fibriform material and particulate matter. The fibriform material (Figures 2A-2) had obvious sags and crests, which was similar to the structure of dietary fiber (the main ingredient) in bamboo shoot. Other main ingredients in bamboo shoot were protein; thus, the morphology of the particulate matter (Figures 2A-4) might be related to the ingredient. The bamboo shoot powder showed clear absorption peaks at 3280 (OH), 2929 (CH_2_), 1629 (C = O), 1395 (C-H), 1045 (C-O), and 886 (C-O-C) cm^–1^ (Figure 2B). Generally, the cellulose in bamboo is the crystalline components with crystallinity degree of 40–60% (Huang et al., 2019b; Lin et al., 2020). In Figure 2C, it is found that there is no crystalline peak in the XRD pattern of bamboo shoot, indicating the obtained substrate contained the amorphous compositions. This can be explained by the fact that the obtained bamboo shoot is the product of bamboo in the early stage (∼2 months), which cannot endow the cellulose in bamboo shoot to crystallization.

### Body Weight

To determine whether bamboo shoot powder suppressed HFD-induced body weight gain, three groups (LFC, HFD, and HFBSP) of mice were separately fed for 7 weeks, and the results are shown in Figure 3.

There was no difference in body weight among the three diet groups at the start of the experiment (day 0), and the body weight between the three groups changed little after the mice were fed for 2 weeks. At week three, the body weight of the HFD mice increased compared with the other two groups. After 7 weeks, the body weight of HFBSP mice was close to that of LFC mice, and the body weight of HFBSP and LFC were both significantly lower than the body weight of HFD mice. Thus, the addition of bamboo shoot powder in the diet of mice can mitigate body weight gain associated with a HFD, indicating that the shoot powder provided health benefits to mice.

### Biochemical Indicators

To investigate the changes of biochemical indicators in mice during the feeding process, the levels of triglycerides (TG), blood glucose (GLU), total cholesterol (CHOL), high-density lipoprotein (HDL-C), and low-density lipoprotein (LDL-C) were compared among the three groups of mice at the end of the experiment (Figure 4). The levels of all five blood lipid indexes were increased in the HFD group compared with the other two groups. TGs are the main component of body fat, and excessive TGs are a direct cause of obesity and fatty liver disease (Lindkvist et al., 2012). The addition of bamboo shoot powder to the mice’s diet could improve the level of TG (*p* < 0.05, vs. HFD group). The GLU level can have a substantial effect on morbidity and mortality; for example, even a slight increase in GLU levels can increase the mortality of patients with COVID-19 (Kesavadev et al., 2021). The GLU level was lower in HFBSP mice than in HFD mice (*p* < 0.01).

Cholesterol CHOL is a key blood lipid index in clinical practice, and high levels of CHOL can lead to diabetes, coronary heart disease, and atherosclerosis (Luo et al., 2019). CHOL levels (3.62 mmol/L, *p* < 0.001) were higher in HFD mice than in LFC mice (2.24 mmol/L). However, the CHOL level of HFBSP mice was low (2.29 mmol/L), which was similar to the CHOL level of LFC mice. Thus, the addition of bamboo shoot powder could decrease the CHOL level when mice consumed a HFD. A high level of LDL-C may lead to atherosclerosis as well as cardiovascular and cerebrovascular diseases, and HFBSP mice had lower LDL-C levels.

Thus, supplementation of fresh Lei bamboo shoot powder reduced the levels of TG, GLU, CHOL, HDL-C, and LDL-C in mice fed a HFD, indicating that the bamboo powder provided health benefits.

### Fat and Liver

**FIGURE 5:**
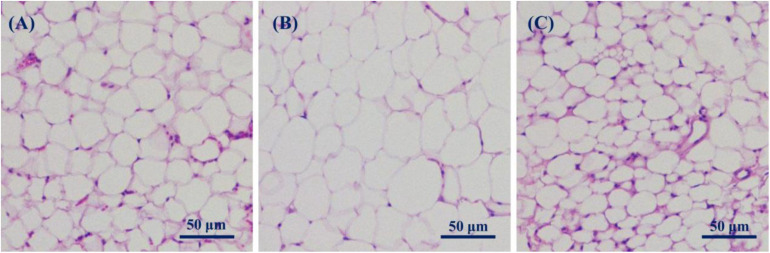
Fat histology images (HE staining). **(A)** LFC; **(B)** HFD; and **(C)** HFBSP.

Significant differences in the pattern and number of mice fat cells were observed among LFC, HFD, and HFBSP mice after they were fed for 7 weeks. Fat histology images with hematoxylin-eosin (HE) staining are shown in Figure 5. The fat cells of HFD mice (Figure 5B) were larger than those of LFC (Figure 5A) and HFBSP mice (Figure 5C). This indicated that HFD mice could lead to obesity, and the addition of bamboo shoot powder to the HFD alleviated obesity, which is consistent with the body weight data shown in Figure 3.

**FIGURE 6:**
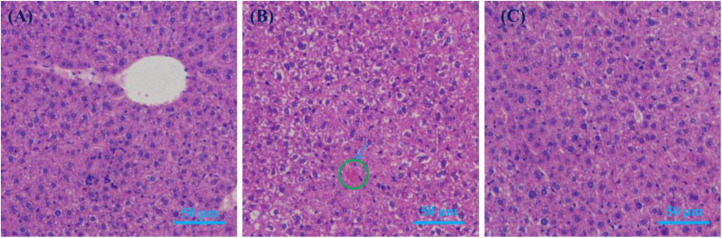
Liver histology images (HE staining). **(A)** LFC; **(B)** HFD; and **(C)** HFBSP.

Liver histology images (HE staining) of hepatocytes are shown in Figure 6. The only noticeable (albeit subtle) difference among the images of the three groups of mice was the presence of a small amount of lipids in the HFD image (Figure 6B). This suggests that HFD mice tended to have fatty livers. Given that no significant obesity was observed in HFBSP mice, the addition of bamboo shoot powder to the HFD might reduce the risk of fatty liver disease.

### Gut Microbiota

**FIGURE 7:**
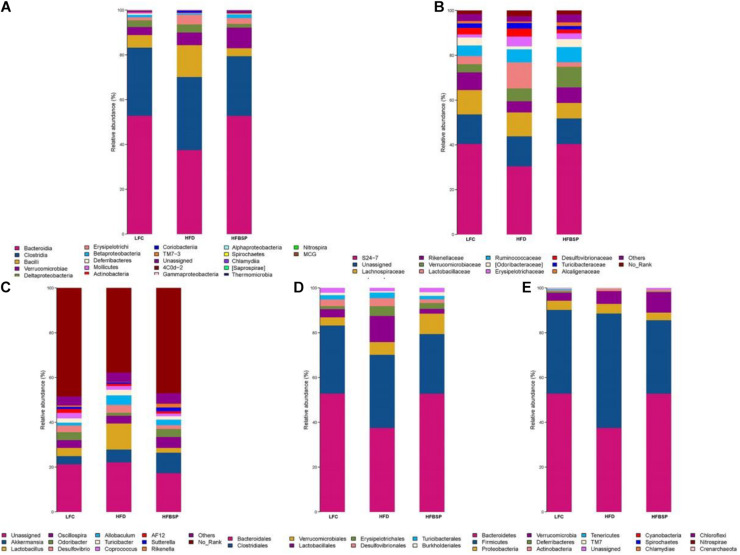
Characterization of the natural gut microbiota of across LFC, HFD, and HFBSP mice (*n* = 10/group) at the class **(A)**, family **(B)**, genus **(C)**, order **(D)**, and phylum **(E)** level.

Species with relative abundances greater than 1% are shown in Figure 7. The relative abundances of different classes of bacteria significantly differed among LFC, HFD, and HFBSP mice (Figure 7A). The abundance of Bacilli was high in HFD mice but low in HFBSP mice. The abundance of Verrucomicrobiae was higher in HFBSP mice than in LFC and HFD mice. The abundances of different families in each group are shown in Figure 7B. The abundance of Lactobacillaceae was significantly higher in HFD mice than in LFC and HFBSP mice, and the abundance of Verrucomicrobiaceae was higher in HFBSP mice than in LFC and HFD mice. Figure 7C shows that the abundances of Lactobacillus and Allobaculum were significantly higher in HFD mice than in LFC and HFBSP mice; the abundance of Akkermansia was higher in HFBSP mice than in LFC and HFD mice.

Figure 7D reveals that the abundance of Lactobacillales was significantly higher in HFD mice than in LFC and HFBSP mice, and the abundance of Verrucomicrobiales was higher in HFBSP mice than in LFC and HFD mice. Figure 7E displays the relative abundance of intestinal microflora at the phylum level. The abundance of Firmicutes was higher in HFD mice than in LFC and HFBSP mice, but the abundance of Bacteroidetes was relatively low; the abundance of Verrucomicrobia was high in HFBSP mice.

Previous studies have shown that there is a relationship between obesity and intestinal flora composition. The abundance of Bacteroides has often been observed to be low in obese patients. However, Bacteroides increased in obese patients during weight loss under a low-fat diet, a finding that is consistent with the body weight data shown in Figure 3. Lactobacillus acidophilus and Clostridium difficile are associated with insulin resistance. Lactobacillus is positively correlated with fasting blood glucose and blood HbA1c levels, whereas C. difficile is negatively correlated with these indicators. Bacteroides, Akkermansia, and other bacteria are known to relieve obesity (Precup and Vodnar, 2019). Thus, the addition of bamboo shoot powder to a HFD could improve gut microbiota and alleviate obesity in mice.

## Conclusion

A bamboo shoot powder was prepared from fresh *Phyllostachys praecox* shoots with a 45.65% dietary fiber content (cellulose, hemicellulose, and lignin). At the end of the 7-week diet experiment, the body weights of HFD mice were higher compared with LFC and HFBSP mice, indicating that this powder could help maintain the weight of HFD mice. The shoot powder supplement could also improve the levels of TG, GLU, CHOL, HDL-C, and LDL-C of HFD mice. In addition, the fat cells of HFD mice were larger than those of LFC and HFBSP mice, which reflected weight gain, and the addition of shoot powder might reduce the fatty liver disease risk of HFD mice. The shoot powder might also improve the gut microbiota profile of HFD mice. Thus, the developed shoot powder could be used as a natural additive that broadens the applications of bamboo shoots in the food industry.

## Data Availability Statement

The raw data supporting the conclusions of this article will be made available by the authors, without undue reservation.

## Ethics Statement

The animal study was reviewed and approved by the Institutional Animal Care and Use Committee (IACUC) of Nanjing Medical University. Written informed consent was obtained from the owners for the participation of their animals in this study.

## Author Contributions

JY, YP, and HY did the experiments and analyzed experimental data. JY and LW designed the research and wrote the manuscript. All authors contributed to the article and approved the submitted version.

## Conflict of Interest

The authors declare that the research was conducted in the absence of any commercial or financial relationships that could be construed as a potential conflict of interest.
